# Beyond the Fragile X protein: neighborhood characteristics explain individual differences in IQ and adaptive behaviors of Fragile X syndrome

**DOI:** 10.3389/fpsyt.2025.1636987

**Published:** 2025-09-18

**Authors:** Walker S. McKinney, Austin Corsmeier, Ashley Dapore, Christina Gross, Kelli C. Dominick, Craig A. Erickson, Lauren M. Schmitt

**Affiliations:** ^1^ Department of Behavioral Medicine and Clinical Psychology, Cincinnati Children’s Hospital Medical Center, Cincinnati, OH, United States; ^2^ Loewenberg College of Nursing, University of Memphis, Memphis, TN, United States; ^3^ Division of Child and Adolescent Psychiatry, Cincinnati Children’s Hospital Medical Center, Cincinnati, OH, United States; ^4^ Department of Pediatrics, University of Cincinnati College of Medicine, Cincinnati, OH, United States; ^5^ Division of Neurology, Cincinnati Children’s Hospital Medical Center, Cincinnati, OH, United States; ^6^ Department of Psychiatry and Behavioral Neuroscience, University of Cincinnati College of Medicine, Cincinnati, OH, United States; ^7^ Phelan-McDermid Syndrome Foundation, Osprey, FL, United States

**Keywords:** Fragile X syndrome, social determinants of health, child opportunity index, neurodevelopmental disabilities, intellectual disability, autism spectrum disorder

## Abstract

**Background:**

Fragile X syndrome (FXS) is the most common inherited cause of intellectual disability and is caused by reduced or absent Fragile X messenger ribonucleoprotein (FMRP). Cognitive and adaptive skills widely vary among individuals with FXS, and these individual phenotypic differences are not fully accounted for by individual differences in FMRP expression. Social-environmental factors, including social determinants of health, may help further explain these individual differences, but these environmental factors have been under-studied in FXS.

**Methods:**

175 participants with FXS (123 males; age range: 4–72 years) completed the Stanford-Binet, Fifth Edition to estimate IQ and a blood draw to quantify peripheral FMRP levels. Caregivers from a subset of participants also completed the Vineland Adaptive Behavior Scales. Neighborhood-level social-environmental information was extracted by linking participants’ home addresses to rankings of neighborhood resources (e.g., household income, pollution, healthcare access) from the Child Opportunity Index (COI). We calculated the unique variance in IQ and adaptive behaviors accounted for by these neighborhood-level social-environmental factors from the COI while covarying for FMRP expression.

**Results:**

Even after accounting for individual differences in FMRP, numerous neighborhood factors were associated with greater IQ in males with FXS, including social resources and indicators of healthcare access. Different social-environment factors were associated with stronger adaptive skills in males with FXS, including economic and educational resources. Almost no neighborhood factors were associated with clinical outcomes in females.

**Discussion:**

Our finding of stronger links between neighborhood resources and clinical outcomes in males with FXS is consistent with previous work and may reflect increased reliance on social-environmental supports in males who typically have more significant intellectual and adaptive deficits than females. Consistent associations between greater social resources, higher IQ, and stronger adaptive skills suggest social support (e.g., social cohesion, resource and knowledge sharing) may be a particularly salient target for intervention. Associations between economic resources and adaptive communication skills also highlight the benefits of targeted economic supports for families affected by FXS. Together, our findings underscore the role of social determinants of health as key contributors to individual differences and the importance of considering these factors in clinical studies of FXS.

## 1 Introduction

Fragile X Syndrome (FXS) is the most common heritable cause of intellectual disability (ID) and single-gene cause of autism spectrum disorder (ASD) (1). FXS is caused by an expansion in the Fragile X messenger ribonucleoprotein 1 (*FMR1*) gene. This expansion results in methylation of the gene which leads to subsequently drastically reduced or absent Fragile X messenger ribonucleoprotein (FMRP). FMRP is critical for typical brain development, and reduced FMRP expression is thought to cause the intellectual disability characteristic of FXS (2). The degree of intellectual impairment differs between affected males (mean IQ = 41, SD = 21) and females (mean IQ = 78, SD = 18), with females typically having higher IQ and stronger adaptive behaviors due to the presence of a second unaffected X chromosome and random X-inactivation (3, 4). Despite the single-gene nature of the disorder, there are immense phenotypic differences from patient to patient, even among males. Some individuals with FXS use spoken language, perform self-care tasks independently, and hold a part- or full-time job, whereas others require significant support in each of these areas and require 24/7 supervision for safety. Yet, these robust differences are only partially accounted for by individual differences in FMRP expression (4–7).

Building on research documenting a wide range of cognitive outcomes in FXS (5, 8–10), we recently proposed a framework of a downshifted, but near normally distributed, range of IQ scores in individuals with FXS (4). Only 5% and 20% of the variance in IQ in our sample of males and females with FXS, respectively, was accounted for by individual differences in peripheral FMRP (11). Even when separately examining males with FXS who had no detectable peripheral FMRP, IQ scores ranged from approximately -20 to 70 (using the Deviation IQ method; Sansone et al., 2014). This work underscores the important, but not singular, role that variation in FMRP expression plays in shaping the cognitive development of individuals with FXS. At a group level, lower levels of FMRP drive reduced IQ and intellectual disability; however, at an individual level, differences in FMRP expression provide an incomplete explanation for large phenotypic differences.

What drives the phenotypic variability among patients with FXS besides differences in FMRP expression? Multiple studies give an unsurprising answer: many of the same factors that contribute to individual differences in the general population (13, 14) also drive phenotypic variation in individuals with FXS. In addition to appreciable differences linked to family genetics (15), social-environmental factors are critical for explaining clinical heterogeneity in syndromic IDs more broadly but remain understudied (16). Key social-environmental factors impacting IQ in FXS include parenting factors (17, 18), home enrichment (19), family stress (20), and parental education (21). Still, findings from these earlier studies are not equivocal, and associations between IQ and social-environmental factors may vary by sex and cognitive domain (verbal vs. nonverbal skills) in FXS (8, 22).

Although some research has identified social-environmental factors that may drive behavioral differences in FXS, many associations between developmental/cognitive outcomes and social-environmental factors observed in studies of the general population have yet to be explored in FXS. We have used a social determinants of health framework (SDOH: “the conditions in the environments where people are born, live, learn, work, play, worship, and age that affect a wide range of health, functioning, and quality-of-life outcomes and risks” (23)) to systematically identify social-environmental factors that have been studied and those that remain to be examined in syndromic IDs (16). For example, unexplored SDOH in FXS include housing factors (e.g., quality, density, stability), school factors (e.g., teaching experience, extracurricular enrichment opportunities), neighborhood safety, environmental conditions (e.g., pollution, air and water quality, extreme weather conditions), social cohesion, healthcare factors (e.g., insurance, provider proximity and density), and employment opportunities.

Not all these factors and the mechanisms through which they impact development are fully understood, but three recent studies in the general population highlight broad trends. First, Putnick and colleagues demonstrated that neighborhood-level resources (as measured by the Child Opportunity Index, or COI), especially healthcare and economic resources, are longitudinally associated with positive developmental outcomes in infants and toddlers (14). Second, Reed and Hillman identified positive associations between neighborhood economic resources (as measured by the Area Deprivation Index) and intelligence in older adults (24). Together, these studies suggest the developmental impact of SDOH begins in infancy and continues into adulthood. Third, Gornik and colleagues found that these neighborhood resources (COI) accounted for substantially greater variance in verbal skills relative to nonverbal skills, suggesting SDOH may disproportionately impact verbal IQ relative to nonverbal IQ (13). These findings underscore the value of leveraging broad, neighborhood-level measures to identify key SDOH that shape developmental and cognitive outcomes across the lifespan, but these neighborhood-level outcomes have not yet been examined in FXS. Characterization of these and other factors in FXS is critical to identifying targets for family- and systems-level (e.g., public policy) interventions that will ultimately improve quality of life for patients and families.

The present study examined associations between a comprehensive set of neighborhood-level SDOH, characterized using the Child Opportunity Index (COI 3.0) (25), IQ, and adaptive behaviors (i.e., practical daily living skills individuals use to function independently across settings) in a large sample of individuals with FXS. Given our interest in identifying salient factors contributing to variation above and beyond FMRP in FXS, we examined these associations while accounting for peripheral FMRP expression. Consistent with findings in typically developing individuals (13) and individuals with other syndromic IDs (26, 27), we hypothesized that associations between SDOH and behavior would be stronger for verbal (verbal IQ, adaptive communication skills) relative to nonverbal skills.

## 2 Materials and methods

### 2.1 Participants

Participants included patients with FXS at the Cincinnati Fragile X Research and Treatment Center seen for a clinical research visit between 2014 and 2024 as part of multiple past and ongoing studies of FXS (age: M = 21.8 years, range = 4.7 - 72.1 years; N = 123 males; N = 52 females). Participants were included if they had at least one visit during which they completed the Stanford-Binet, Fifth Edition and a blood draw. A subset of participants (N = 28) provided data at multiple independent visits. Repeated visits occurred either because (1) participants were enrolled in the FORWARD FXS natural history study which entails longitudinal follow-up visits, or (2) participants were enrolled in multiple studies (e.g., a participant completed the Stanford-Binet and a blood draw in 2020 as part of Study 1, then returned in 2022 to complete the Stanford-Binet and a blood draw as part of Study 2). There were 175 unique participants providing data across 206 unique visits. Participants providing data at multiple visits were not significantly different from those providing data only at one visit in terms of age, sex, race, ethnicity, FMRP, full-scale IQ, nor total COI scores (all *p* >.05).

All participants had a confirmed diagnosis of FXS, defined as having the full *FMR1* mutation (>200 CGG repeats), confirmed via past testing results available in a participant’s medical record or via Southern Blot and/or PCR conducted in collaboration with the Molecular Diagnostic Laboratory at Rush University. Full demographic details are reported in **Table 1**.

**Table 1:** Demographics.

	M (SD) or N (%)	
	Males (N = 123)	Females (N = 52)
Age (years)	21.9 (12.4)	21.4 (14.1)
FMRP (pico molar)*	1.5 (3.0)	21.5 (7.7)
Race		
Asian-American/Pacific Islander	1 (0.8%)	2 (3.9%)
Black	3 (2.4%)	1 (1.9%)
Multiracial	1 (0.8%)	0 (0%)
White	118 (95.9%)	49 (94.2%)
Ethnicity		
Hispanic or Latino	6 (5.0%)	2 (3.8%)
Non-Hispanic or Latino	115 (95.0%)	50 (96.2%)
Caregiver 1 Highest Education		
High school graduate	12 (9.8%)	2 (3.8%)
Some college	23 (18.7%)	6 (11.5%)
College	29 (23.6%)	15 (28.8%)
Postgraduate	26 (21.1%)	15 (28.8%)
Missing data	33 (26.8%)	14 (26.9%)
Caregiver 2 Highest Education		
High school graduate	12 (9.8%)	4 (7.7%)
Some college	17 (13.8%)	8 (15.4%)
College	29 (23.6%)	14 (26.9%)
Postgraduate	23 (18.7%)	10 (19.2%)
Missing data	42 (34.1%)	16 (30.8%)
Household Income		
≤ $20,000	7 (5.7%)	5 (9.6%)
$20,001 - $40,000	5 (4.1%)	1 (1.9%)
$40,001 - $60,000	5 (4.1%)	4 (7.7%)
$60,001 - $90,000	19 (15.4%)	8 (15.4%)
≥ $90,001	47 (38.2%)	21 (40.4%)
Missing data	40 (32.5%)	13 (25.0%)
Stanford-Binet 5		
Full-Scale Deviation IQ*	41 (21)	78 (18)
Verbal Deviation IQ*	41 (21)	79 (18)
Nonverbal Deviation IQ*	41 (23)	76 (21)
Vineland-3		
Adaptive Behavior Composite*	45 (19)	81 (16)
Communication*	37 (19)	77 (22)
Daily Living Skills*	48 (21)	85 (15)
Socialization*	49 (22)	85 (17)
COI		
Overall	66 (26)	66 (26)
Social and economic	64 (27)	64 (27)
Health and environment	63 (24)	63 (25)
Education	68 (26)	68 (25)

Caregiver 1 and Caregiver 2 labels reflect the primary and secondary caregiver (e.g., mother and father, mother and grandparent); For participants represented across multiple visits, only values for their most recent visit are reported for this table. * Denotes males vs. female comparison significant at *p* <.05; Abbreviations: M, mean; SD, standard deviation; N, sample size; FMRP, fragile X messenger ribonucleoprotein; COI, Child Opportunity Index 3.0.

All studies were approved by the Cincinnati Children’s Hospital Medical Center Institutional Review Board. Parents, caregivers, or other legally authorized representatives provided written consent for participants younger than 18 years of age or for participants who were unable to provide consent due to limited informed decision-making capacity stemming from their intellectual disability. Participants provided their written consent when possible and otherwise provided verbal assent when possible.

### 2.2 Procedures

#### 2.2.1 Stanford-Binet, Fifth Edition

All participants completed the Stanford-Binet, Fifth Edition (SB-5) administered by a licensed clinical psychologist, postdoctoral fellow, or clinical research coordinator. The full version of the SB-5 was completed at 167 visits (N = 149 unique participants), and the routing/abbreviated form of the SB-5 was completed for all remaining visits and participants. Deviation scores for the SB-5 full-scale IQ (FSIQ), abbreviated IQ (ABIQ), verbal IQ (VIQ), and nonverbal IQ (NVIQ) scales were calculated using previously reported methods validated for use in FXS to minimize floor effects common in this population (4, 12). To allow comparison of routing Verbal Knowledge performance with VIQ and routing Nonverbal Fluid Reasoning performance with NVIQ using the same standard scale, deviation z-scores for each routing subtest were transformed to standard scores. Due to the strong correlations between FSIQ and ABIQ in our sample (*r* = .94, *p* <.001), VIQ and Verbal Knowledge performance (*r* = .94, *p* <.001), and NVIQ and Nonverbal Fluid Reasoning performance (*r* = .88, *p* <.001), the routing SB-5 ABIQ, Verbal Knowledge, and Nonverbal Fluid Reasoning were substituted for FSIQ, VIQ, and NVIQ, respectively, when a full administration was not available for a given visit. In addition to the strong correlations between FSIQ and ABIQ observed in this sample, this decision was also made in consideration of previous findings demonstrating that the predictive validity of ABIQ in estimating FSIQ is strong in youth with neurodevelopmental disabilities when the scatter between the routing subtests is small (especially < 4) (28), as was the case in this sample (absolute difference in scaled scores: males: M = 0.6, SD = 1.2; females: M = 2.4, SD = 2.1). Participants who completed a full SB-5 were not significantly different from those who only completed the routing/abbreviated form in terms of age, sex, race, ethnicity, FMRP, IQ (i.e., FSIQ from full SB-5 group, ABIQ from routing only group), nor total COI scores (all *p* >.05). We analyzed standard scores for deviation FSIQ, VIQ, and NVIQ.

#### 2.2.2 Vineland adaptive behavior scales

A subset of participants’ caregivers (N = 150 visits; N = 127 unique participants) also completed the Vineland Adaptive Behavior Scales, Third Edition, Comprehensive Interview Form (Vineland-3) (29), a measure of adaptive functioning. All caregivers completing the Vineland completed this same examiner-administered version, administered by either a licensed psychologist or supervised postdoctoral fellow/trained research coordinator. Interrater reliability was sustained via quarterly interrater reliability meetings to discuss scoring questions and review sample responses and scores. Because our dataset reflects participants across more than a dozen studies and protocol differences across studies, not all participants have data available for the Vineland. Participants with Vineland data did not significantly differ from those without Vineland data on sex, race, ethnicity, FMRP, full-scale IQ, nor total COI scores (*p*’s >.05). Participants with Vineland data were significantly younger (M = 18.5 years) than those without Vineland data (M = 28.1 years; *t*(75.70) = 4.245, *p* <.001). This is driven by protocol differences, where some studies providing data for these results were for children only and required completion of the Vineland, while some studies that allowed adult participation did not require completion of the Vineland. We analyzed standard scores from the Adaptive Behavior Composite (ABC) and the three Vineland-3 domains: Communication, Daily Living Skills, and Socialization.

#### 2.2.3 Fragile X protein

All participants completed a blood draw. The amount of Fragile X protein (FMRP) was quantified from dried blood spots using our previously described Luminex-based immunoassay (11).

### 2.3 Geocoding and extraction of the Child Opportunity Index

Participants’ self-reported addresses at the time of clinical testing were extracted from our hospital’s electronic health record (EHR). Addresses were geocoded using the publicly available US Census Geocoder (https://geocoding.geo.census.gov/geocoder**/** ) to extract 2010 census tract Federal Information Processing Standards (FIPS) codes. “Non-exact” and “tied” address matches were manually resolved and verified (e.g., “1234 Appletree Lane” may match to two US Census addresses: “1234 Apple Tree Lane” and “1234 Appletree Lane”). Remaining non-matches (~1.9% of addresses) occurred due to the home being recently constructed, demolished, or located in an extremely rural area. For these addresses, the nearest neighboring home address was used as input to the Census Geocoder as an approximation of the participant’s home address, which enabled systematic and accurate extraction of the census tract for the original address. This form of address interpolation is well-established for projects that rely only on coarse, tract-level matching as in the present study (30).

Census tracts for participant addresses were linked to the Child Opportunity Index 3.0 (COI) (25). The COI is a publicly available dataset providing standardized information about neighborhood characteristics related to health outcomes (i.e., social determinants of health). The COI 3.0 includes an overall composite score (“total neighborhood opportunity”), 3 domains (social and economic, health and environment, education), and 14 subdomains based on 44 component indicators (**Table 2**). Component indicators are weighted based off the strength of their correlation with key socioeconomic and health outcomes. COI outcomes are rank-ordered percentiles ranging from 1 (least amount of opportunity) to 100 (most amount of opportunity). Because our data encompasses patients living across the United States, we used the nationally-ranked COI.

**Table 2:** Child Opportunity Index domains, subdomains, and indicators.

COI domain/subdomain	Indicators
Total neighborhood opportunity	All domains/subdomains
Total economic opportunity	All economic subdomains
Economic equity	Adults with advanced degrees; Very high-income households; Adults without high school degrees; Very low-income households
Employment	Employment rate; High-skill employment rate; Full-time year-round earnings
Economic resources	Median household income; Poverty rate; Public assistance rate
Housing	Broadband internet access; Crowded housing
Social resources	Mobility-enhancing friendship networks; Single-parent families; Non-profit organizations
Wealth	Homeownership rate; Aggregate home values; Aggregate capital income; Aggregate real estate taxes
Total health and environmental opportunity	All health/environment subdomains
Healthy air/water	Airborne microparticles; Ozone concentration; Industrial pollutants; Hazardous waste dump sites
Healthcare resources	Health-related non-profits; Health insurance coverage
Safety	Community safety-related non-profits; Vacant housing
Healthy environments (i.e., nutrition/weather)	Healthy food retailer density; Extreme heat exposure; NatureScore; Walkability
Total educational opportunity	All educational subdomains
Early childhood education	Private pre-K enrollment; Public pre-K enrollment
Elementary education	Reading and math test scores; Reading and math test score growth; Poverty-adjusted reading and math test scores
High school/college	Advanced Placement course enrollment; College enrollment in nearby institutions; High school graduation rate
Educational quality	Adult educational attainment; Child enrichment-related non-profits; Teacher experience; School poverty

COI, Child Opportunity Index 3.0.

### 2.4 Statistical analyses

Linear mixed effects models were used for all analyses. Dependent outcomes were either IQ (FSIQ, VIQ, NVIQ) or adaptive behavior performance (Adaptive Behavior Composite and the Communication, Daily Living Skills, and Socialization subdomains). Independent variables included peripheral FMRP, chronological age (a covariate of no interest), and the COI ranking. Separate models were run for COI rankings of total neighborhood opportunity, the 3 COI domains, and the 14 COI subdomains. Models were run separately for males and females due to known sex differences in FMRP expression and previously reported sex differences in the association between environmental factors and clinical outcomes in FXS (15, 19). Participant/subject was the random effect in all models to account for participants who were tested at multiple visits. The variance accounted for by each fixed effect in our figures reflects the coefficient of determination (semi-partial R squared) calculated using methods from Nakagawa and Schielzeth (31) using the *r2glmm* R package (32). Unless otherwise noted in the text, the direction of all associations was positive (e.g., more positive social-environmental exposures related to greater IQ). Additional tertiary models (associations between FMRP and IQ, FMRP and adaptive behaviors, FMRP and COI) are presented in the **Supplementary Materials** document. Due to the hypothesis-generating and exploratory nature of this study as well as the limited work in this area, we did not correct for multiple comparisons.

All statistical analyses were conducted using R version 4.3.1 (33). Linear mixed effects models were constructed using the *lme4* R package (34). All data was visualized using the *ggplot2* R package (35).

## 3 Results

### 3.1 Neighborhood descriptive characteristics

Participants came from 23 different states and 90 different counties across the United States, reflecting the national makeup of patients seen in our clinic.

**Figure 1:**
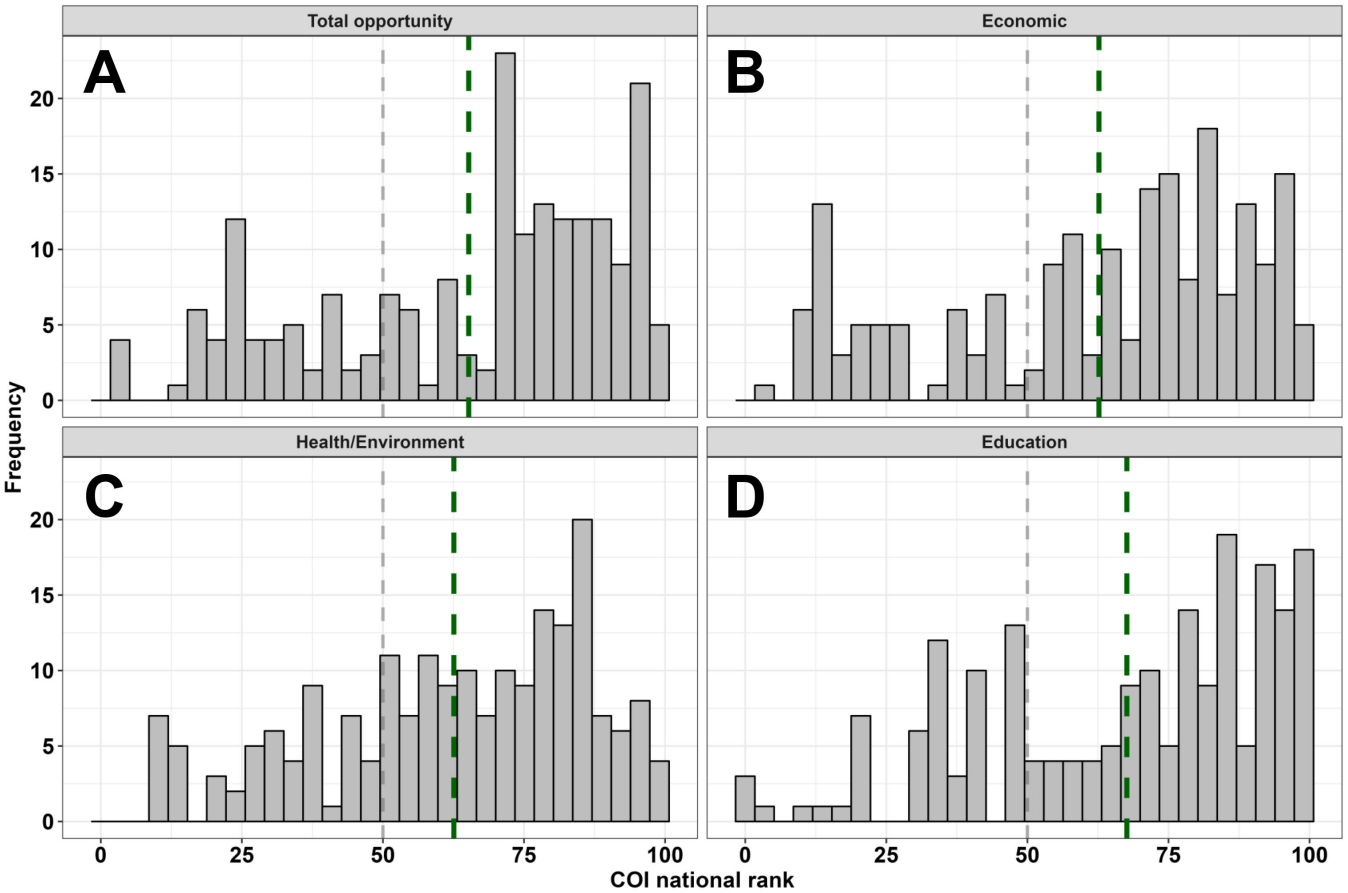
Sample distribution of neighborhood opportunities for the total COI **(A)** and three domains **(B-D)**. The dashed grey line reflects a COI rank of 50 (i.e., general population, national mean), and each dashed green line reflects the sample mean for each respective panel’s domain.

**Figure 2:**
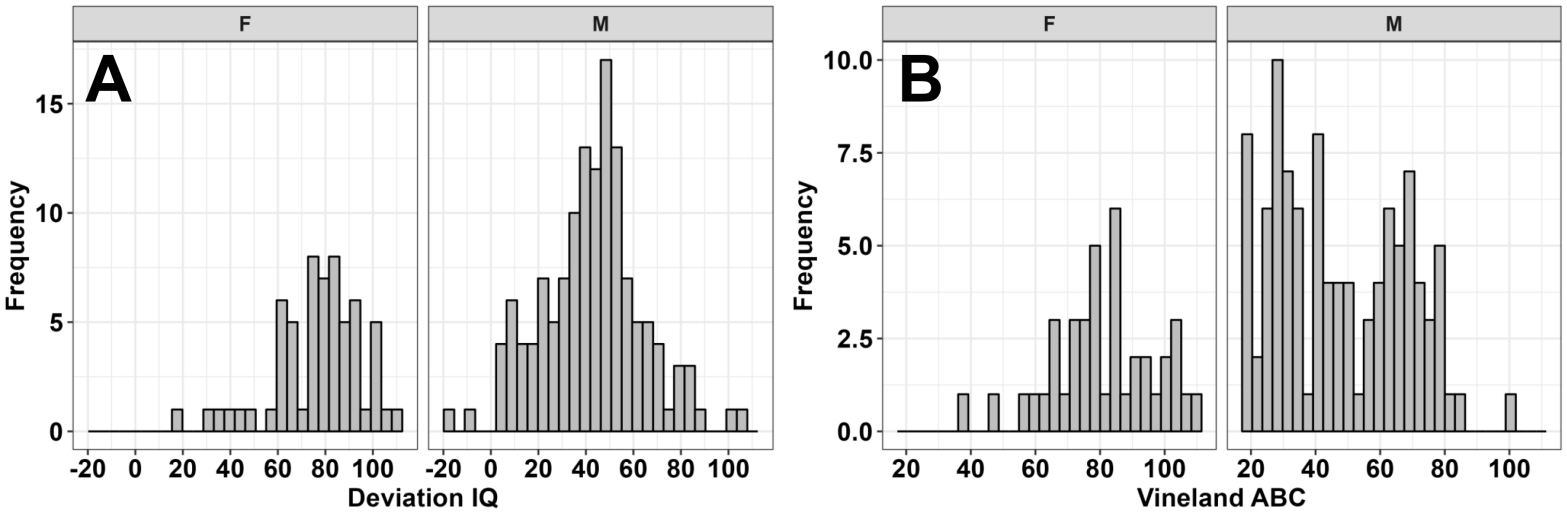
Sample distribution of deviation full-scale IQ **(A)** and Vineland-3 Adaptive Behavior Composite scores **(B)** for females (F) and males (M).

Overall neighborhood opportunity as measured by the COI widely varied among the full sample, but skewed towards higher opportunity areas (M = 66; SD = 26; Range: 3-99). The distribution of neighborhood opportunities was similar across the social and economic (M = 64; SD = 27; Range: 10-99), health and environment (M = 63; SD = 24; Range: 9-100), and education (M = 68; SD = 26; Range: 1-100) COI domains (**Figure 1**). IQ and adaptive behavior sample distributions are depicted in **Figure 2**. Males and females did not differ on the COI (*p*’s >.89).

### 3.2 Associations between neighborhood characteristics and IQ

#### 3.2.1 Males

**Figure 3:**
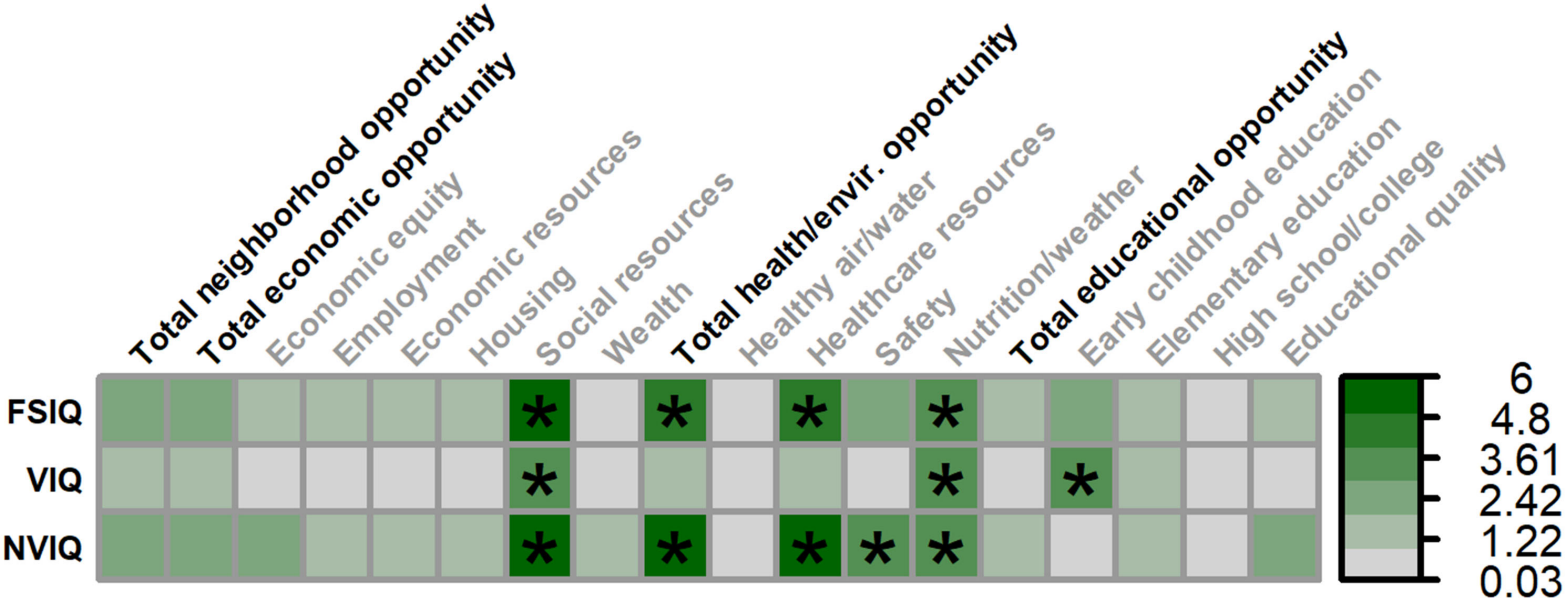
Matrix depicting the percent variance (color gradient) in full-scale IQ (FSIQ), verbal IQ (VIQ), and nonverbal IQ (NVIQ) accounted for by each COI domain and subdomain in males. Values reflect variance after accounting for FMRP expression and age. Bolded factors denote COI domains, and un-bolded factors denote COI subdomains. Asterisks (*) denote p <.05.

**Figure 4:**
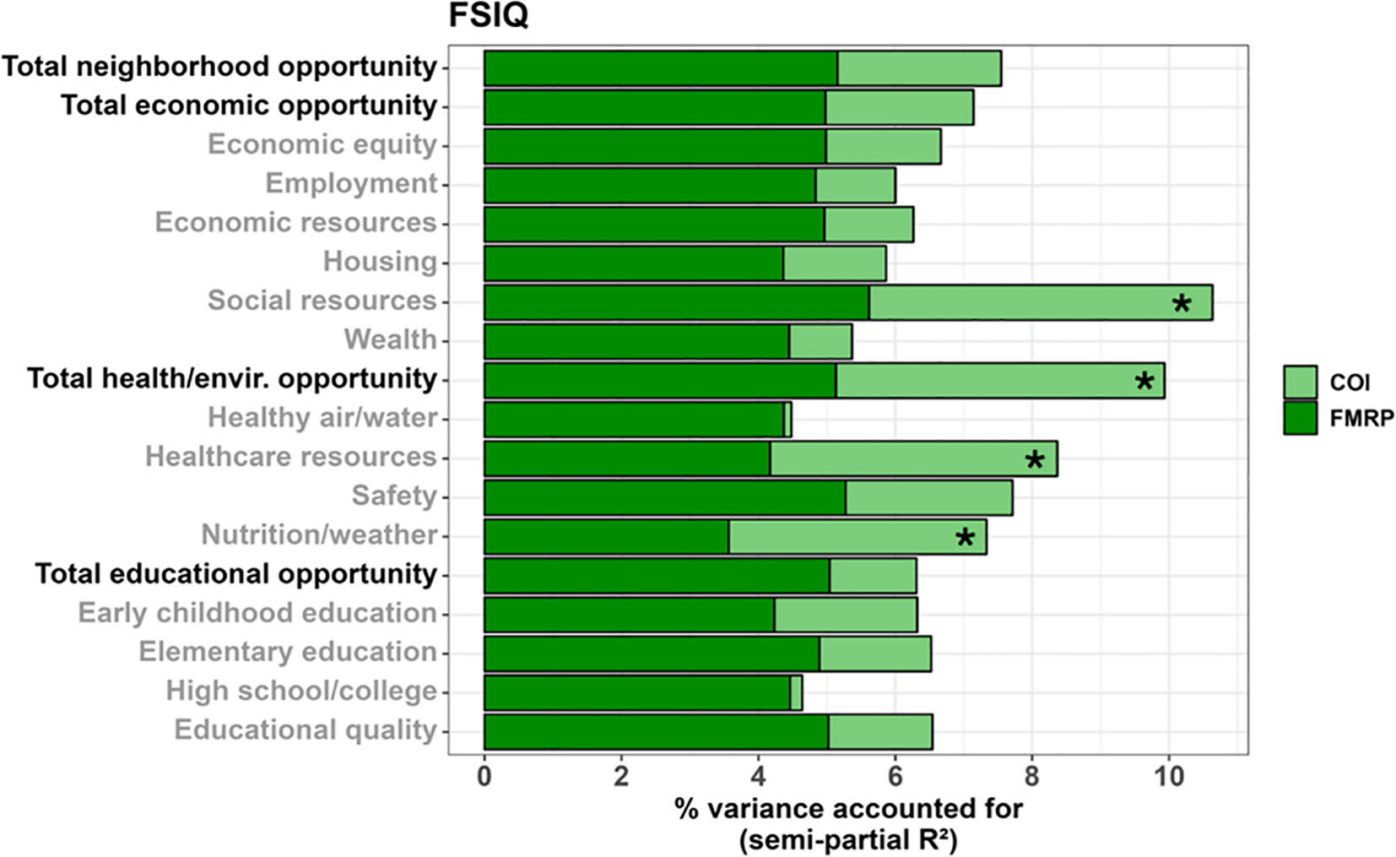
Stacked bar chart depicting the percent variance in full-scale IQ (FSIQ) accounted for by FMRP (dark green) and each COI domain and subdomain (light green) in males. Values reflect variance after accounting for age. Bolded factors denote COI domains, and un-bolded factors denote COI subdomains. Asterisks (*) denote a COI domain/subdomain significantly associated with FSIQ at p <.05.

Multiple SDOH, as measured by the COI, were associated with greater full-scale IQ in males (**Figures 3**, **4**), including: social resources (*F_(1,115.797)_* = 6.179, *p* = .014, 5.02% unique variance), total health/environment opportunities (*F_(1,131.991)_* = 7.052, *p* = .009, 4.80% unique variance), healthcare resources (*F_(1,130.860)_* = 6.325, *p* = .013, 4.20% unique variance), and healthy environments (i.e., nutrition and weather; *F_(1,119.308)_* = 4.735, *p* = .032, 3.77% unique variance).

Multiple SDOH were also associated with greater verbal IQ in males, including: social resources (*F_(1,120.255)_* = 4.798, *p* = .030, 3.94% unique variance), healthy environments (i.e., nutrition and weather; *F_(1,122.545)_* = 4.220, *p* = .042, 3.35% unique variance), and early childhood education (*F_(1,130.300)_* = 4.672, *p* = .032, 3.47% unique variance).

A similar set of SDOH were associated with greater nonverbal IQ in males, including: social resources (*F_(1,106.510)_* = 6.858, *p* = .010, 5.29% unique variance), total health/environment opportunities (*F_(1,132.769)_* = 8.774, *p* = .004, 6.00% unique variance), healthcare resources (*F_(1,134.778)_* = 7.756, *p* = .006, 5.24% unique variance), neighborhood safety (*F_(1,133.590)_* = 4.731, *p* = .031, 3.36% unique variance), and healthy environments (i.e., nutrition and weather; *F_(1,115.752)_* = 4.153, *p* = .044, 3.21% unique variance).

#### 3.2.2 Females

**Figure 5:**
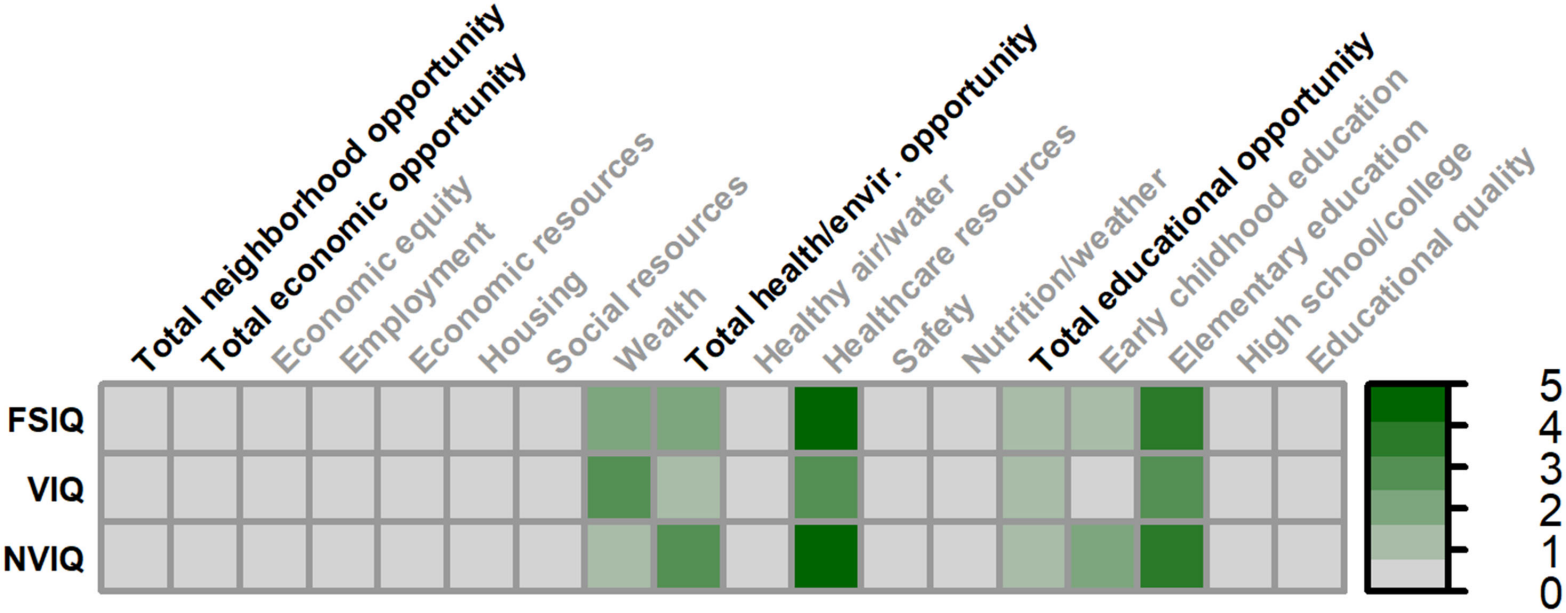
Matrix depicting the percent variance (color gradient) in full-scale IQ (FSIQ), verbal IQ (VIQ), and nonverbal IQ (NVIQ) accounted for by each COI domain and subdomain in females. Values reflect variance after accounting for FMRP expression and age. Bolded factors denote COI domains, and un-bolded factors denote COI subdomains. All associates were non-significant in females.

No domains or subdomains of the COI were associated with full-scale IQ, verbal IQ, or nonverbal IQ in females with FXS (**Figure 5**).

### 3.3 Associations between neighborhood characteristics and adaptive behaviors

#### 3.3.1 Males

**Figure 6:**
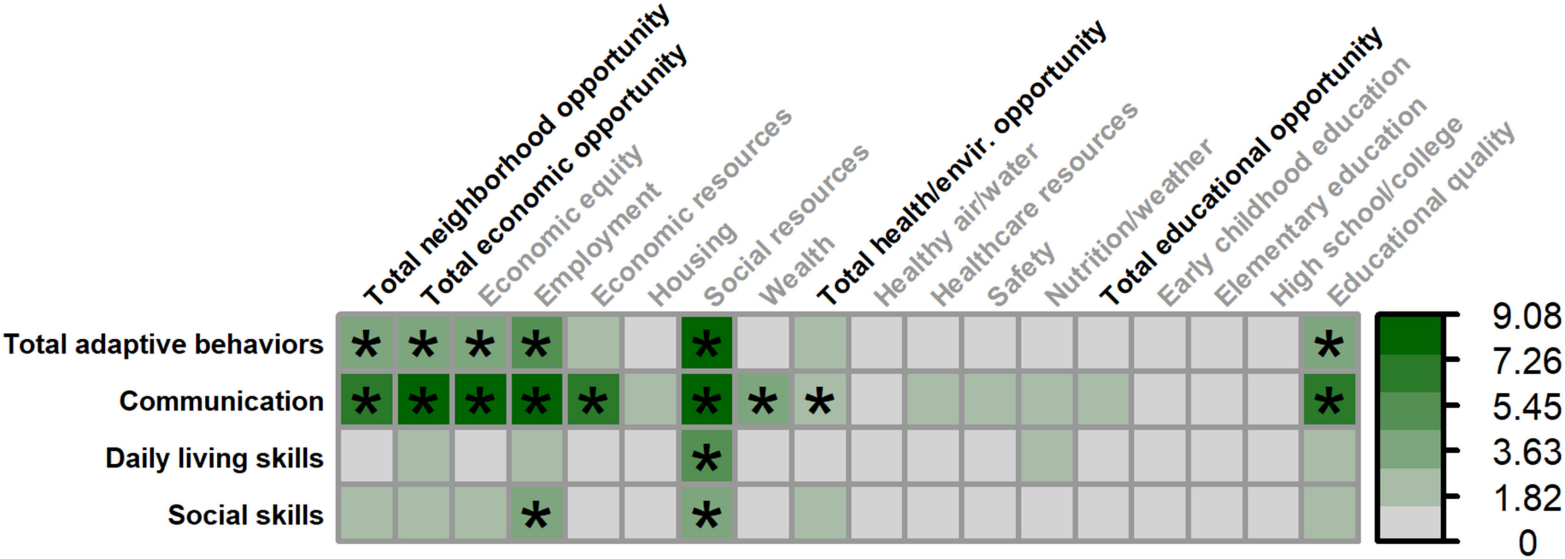
Matrix depicting the percent variance (color gradient) in the Vineland-3 Adaptive Behavior Composite and three subdomains accounted for by each COI domain and subdomain in males. Values reflect variance after accounting for FMRP expression and age. Asterisks (*) denote p <.05.

**Figure 7:**
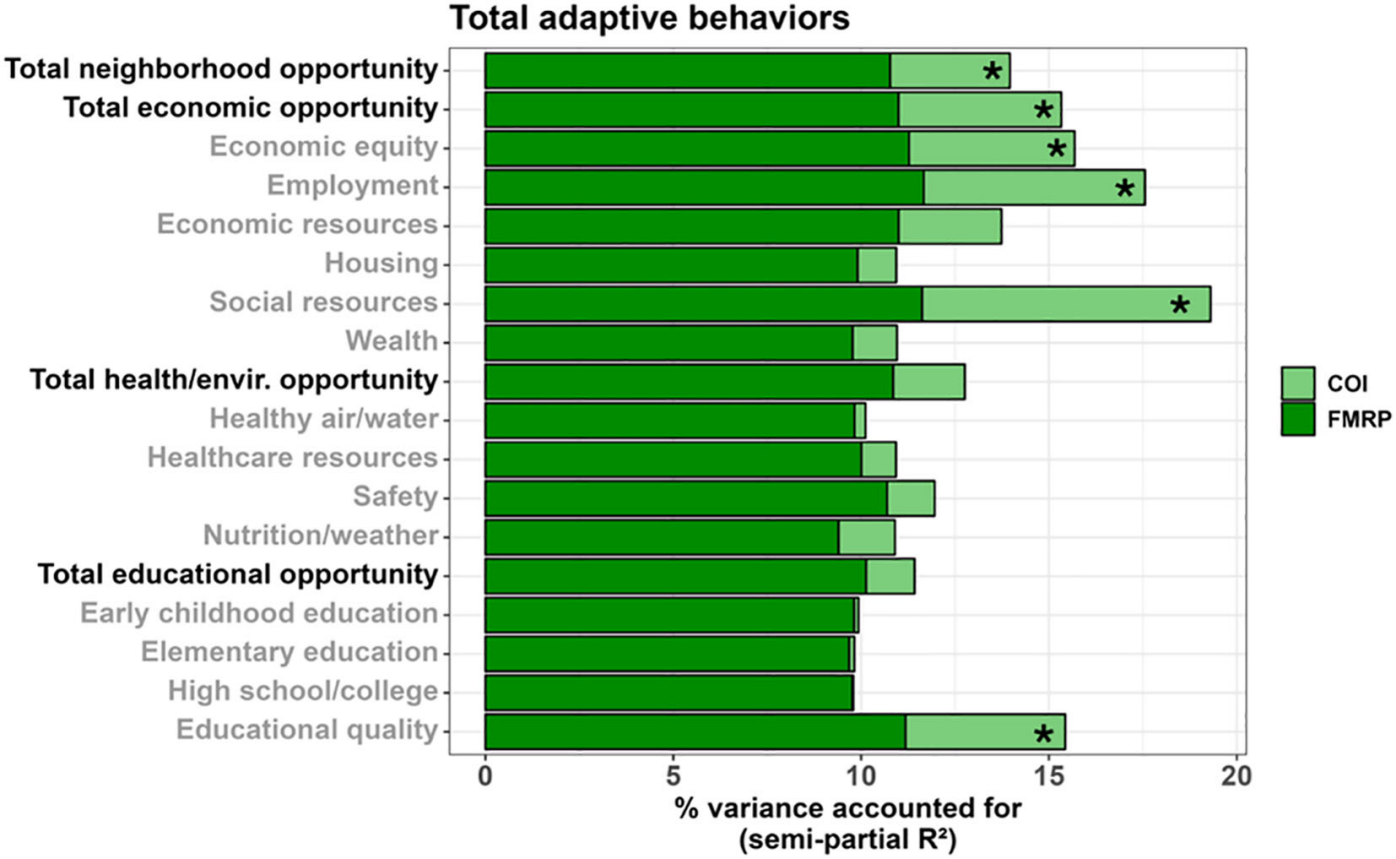
Stacked bar chart depicting the percent variance in total adaptive behaviors (Vineland ABC) accounted for by FMRP (dark green) and each COI domain and subdomain (light green) in males. Values reflect variance after accounting for age. Asterisks (*) denote a COI domain/subdomain significantly associated with the Vineland ABC at p <.05.

Multiple SDOH, as measured by the COI, were associated with greater overall adaptive behaviors (Vineland Adaptive Behavior Composite) in males (**Figures 6**, **7**), including: total neighborhood opportunities (*F_(1,122.734)_* = 4.019, *p* = .047, 3.19% unique variance), total economic opportunities (*F_(1,120.806)_* = 5.424, *p* = .022, 4.34% unique variance), economic equity (*F_(1,124.929)_* = 5.545, *p* = .020, 4.41% unique variance), employment opportunities (*F_(1,116.474)_* = 7.015, *p* = .009, 5.89% unique variance), social resources (*F_(1,122.568)_* = 10.006, *p* = .002, 7.68% unique variance), and educational quality (*F_(1,125.382)_* = 5.367, *p* = .022, 4.25% unique variance).

A similar set of SDOH were associated with greater adaptive communication skills in males, including: total neighborhood opportunities (*F_(1,116.695)_* = 8.508, *p* = .004, 6.49% unique variance), total economic opportunities (*F_(1,115.352)_* = 10.425, *p* = .002, 7.91% unique variance), economic equity (*F_(1,115.960)_* = 11.351, *p* = .001, 8.62% unique variance), employment opportunities (*F_(1,108.014)_* = 10.522, *p* = .002, 8.31% unique variance), economic resources (*F_(1,120.326)_* = 8.113, *p* = .005, 6.18% unique variance), social resources (*F_(1,116.401)_* = 12.122, *p* <.001, 9.08% unique variance), neighborhood wealth (*F_(1,129.544)_* = 4.321, *p* = .040, 3.18% unique variance), total health/environment opportunities (*F_(1,126.832)_* = 3.981, *p* = .048, 2.96% unique variance), and educational quality (*F_(1,115.460)_* = 9.105, *p* = .003, 7.01% unique variance).

Only greater social resources were associated with greater daily living skills in males (*F_(1,122.065)_* = 7.412, *p* = .007, 5.80% unique variance).

Greater adaptive social skills were associated with greater employment opportunities (*F_(1,118.007)_* = 4.869, *p* = .029, 4.16% unique variance) and social resources (*F_(1,124.276)_* = 5.670, *p* = .019, 4.48% unique variance) in males.

#### 3.3.2 Females

**Figure 8:**
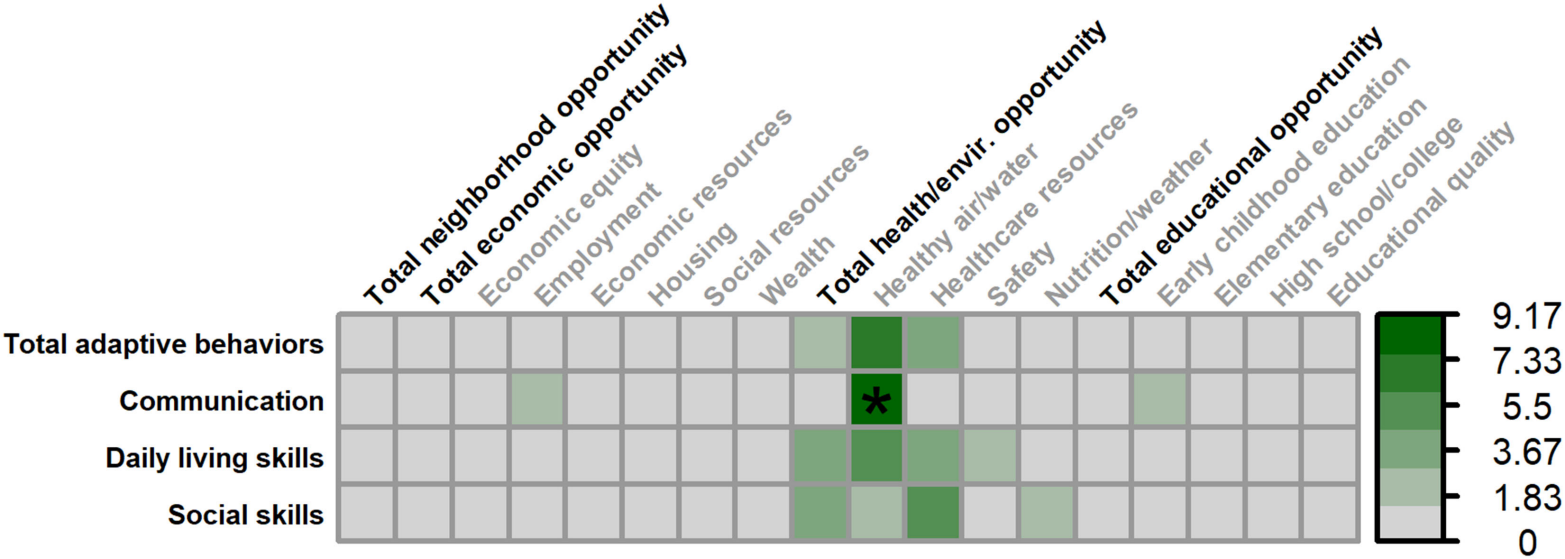
Matrix depicting the percent variance (color gradient) in the Vineland-3 Adaptive Behavior Composite and three subdomains accounted for by each COI domain and subdomain in females. Values reflect variance after accounting for FMRP expression and age. Asterisks (*) denote p <.05.

Greater air and water quality was associated with lower adaptive communication skills in females (*F_(1,38.373)_* = 4.401, *p* = .043. 9.17% unique variance). No other SDOH were associated with adaptive behaviors in female (**Figure 8**).

## 4 Discussion

We examined associations between a comprehensive set of standardized social-environmental metrics and clinical outcomes in FXS. We were particularly interested in the degree to which these factors were related to individual differences in IQ and adaptive behaviors *after* accounting for individual differences in FMRP expression. Our study replicates past work establishing the consistently modest, but not deterministic, role that FMRP plays in shaping functional outcomes in FXS like IQ and adaptive behaviors (4–6, 36) (**Supplementary Results**). We also demonstrate three novel findings that highlight the importance of non-genetic factors in FXS. First, we identify several neighborhood characteristics that partially explain individual differences in IQ and adaptive behaviors in males with FXS, with several factors explaining nearly the same amount of variance accounted for as individual differences in FMRP expression. Second, we demonstrate these associations with social-environmental factors are specific to males with FXS, consistent with past work finding sex-differences in clinical severity and subsequent environmental accommodations in FXS. Last, the strongest associations were between social-economic factors and adaptive behaviors, especially adaptive communication skills, consistent with past work underscoring the distal role that economic resources play in shaping language development. Ultimately, although research clarifying the biological factors that drive phenotypic variation in FXS is critical, we believe our work highlights the equally urgent need to study and intervene on social-environmental factors in FXS and ID more broadly.

### 4.1 Robust environmental-behavioral associations in males, but not females, with FXS

Building on previous ID research (15, 37, 38), we show that several social-environmental factors as measured by the COI (e.g., social resources, access to healthcare, neighborhood walkability) are positively related to IQ in males, but not females, with FXS. For example, these factors explained about 3 to 5% of the unique variance in full-scale IQ among males, similar to the 3-5% of the variance in full-scale IQ accounted for by FMRP in this same sample. A largely distinct set of economic and educational factors were associated with adaptive behaviors; again, these associations were only present in our male participants.

These sex-specific findings echo two previous studies in FXS showing that environmental factors are more strongly related to IQ and adaptive behaviors in males compared to females with FXS (15, 19). Although females with FXS also present with clinical concerns such as anxiety and difficulties with conversational pragmatics (39), cognitive and adaptive skills are typically less severely impacted in females with FXS due to their retaining a second unaffected allele and random X-chromosome inactivation (3, 4). These sex differences in cognitive and adaptive outcomes are illustrated for our sample in **Figure 2**.

We and others have hypothesized that sex-specific environment-behavior associations in FXS are driven by an inherently greater need for environmental accommodations and behavioral supports for more clinically affected males (16, 19). This pattern is reflected in FXS studies finding discrepancies in service utilization between males and females (40), greater median healthcare expenditures in males (41) and more frequent specialty Fragile X clinic attendance in patients with greater symptom severity (42). Although we have not demonstrated causality, this suggests that IQ and adaptive behaviors may be especially malleable in males with FXS when supported by environmental resources and interventions (e.g., early childhood intervention programs, adequate insurance coverage). Longitudinal research will help provide further evidence for this hypothesis and clarify the most salient social-environmental factors to target.

Statistically, this sex-specific pattern also may reflect the relatively greater proportion of variance in clinical outcomes accounted for by FMRP in females compared to males, leaving less unique variance that may be accounted for by social-environmental actors in females. About 38% of the males in our sample had no detectable peripheral FMRP (FMRP = 0), limiting the magnitude of observed associations between FMRP and clinical outcomes in males (i.e., leaving more variance to be accounted for by the COI). We believe analyses of females with FXS *and* ID (i.e., IQ < 70) and more males with partial FMRP expression will clarify if these sex-specific associations are underpinned by differences in clinical severity, differences in FMRP expression, or both. The relatively small number of females with IQ < 70 in the present study (N = 15) precludes our ability to conduct an adequately powered analysis, but future large-scale studies of FXS should consider such an approach to clarify this point. Statistically, sex-specific associations also may reflect the larger number of male participants subsequently driving increased statistical power compared to the smaller group of females for whom the risk of Type II errors is increased.

### 4.2 Which neighborhood factors account for the most phenotypic variation in FXS?

The SDOH most consistently associated with nearly all outcomes was social-economic resources. The COI uses three indicators in the calculation of its social resources subdomain: mobility-enhancing friendship networks (i.e., mixed low- and high-income friendships), multi-parent families, and proximity of non-profit organizations within a given census tract. The first two metrics are weighted the most heavily when calculating COI social resources rankings (weighted 0.90 out of 1.0) and may explain why this association is so robust across outcomes. We hypothesize this pattern is largely due to the positive effects of increased caregiver support and reduced parental stress in families embedded within mobility-enhancing social networks and multi-caregiver households. Mobility-enhancing friendships enable transmission of knowledge and sharing of resources, provide emotional support, and reduce parental stress, leading to a range of positive health and developmental outcomes (43, 44). Similarly, parental stress is lower in married parents relative to single parents, likely due to a combination of enhanced social supports (45) and increased household income among married parents (46). Taken together, both forms of caregiver support can reduce family stress.

The Family Stress Model provides a theoretical framework for the impact of this stress on downstream child development (47). Briefly, family stress, through its direct effects on caregiver-child interactions and the home environment, has detrimental effects on cognitive development, including in patients with FXS (16). This effect has been implicated in correlational studies of FXS: greater parental stress is associated with lower fluid and crystallized intelligence (17) and a fewer number of parent-child interactions (20) critical for child development. Follow-up studies of social cohesion will help further specify the mechanisms through which social supports drive greater child IQ and adaptive skills in families of people with FXS. We are especially interested in studies soliciting the lived experiences and input of family members on unique aspects of social support in FXS, such as the availability of respite care, that reduce family stress and support the development of their children.

### 4.3 Neighborhood factors associated with individual differences in adaptive behaviors

Most neighborhood economic factors, including economic equity (e.g., reduced income disparity), employment opportunities, economic resources (e.g., household income, public assistance), and wealth (i.e., capital assets), were associated with stronger adaptive behaviors in males with FXS. Consistent with our hypotheses, these factors were most strongly associated with adaptive communication skills. This is consistent with research in the general population demonstrating that neighborhood-level opportunities are most strongly associated with language outcomes (13). These findings also are in keeping with research in other IDs, including 22q11.2 deletion syndrome (26) and Down syndrome (27), which have found stronger associations between family socioeconomic status (e.g., proxy measures like parental education) and VIQ compared to NVIQ. Although we did not observe the same pattern for VIQ compared to NVIQ, this may reflect the increased susceptibility of adaptive communication skills, which emphasize the *application* of pragmatic verbal skills across settings (e.g., for reading, writing, telling stories, receiving and giving verbal instructions, etc.) (48), to environmental differences.

We hypothesize that these economic factors so strongly relate to communication skills due to the mediating roles of parental responsivity and home enrichment. Several studies of the general population demonstrate that economic stability enables greater parental responsiveness (49, 50). Responsive parent-child interactions, in turn, are longitudinally associated with greater lexical diversity (21) and expressive and receptive language in FXS (18) and the general population (51). This is likely a bidirectional and cyclical relationship, as children with stronger language elicit more responsive parental interactions (52). Importantly, parental responsiveness is a modifiable factor in parents of youth with IDs (for review, see (60)), underscoring the utility of a multipronged intervention approach targeting both distal economic factors and proximal family factors to improve adaptive communication skills in FXS.

Economic resources also likely enable greater home enrichment that supports language development in FXS, as demonstrated in the general population (53). Consistent with this, home enrichment is associated with adaptive communication skills in FXS (19). Home enrichment encompasses materials and activities that stimulate intellectual growth, such as literacy materials, cognitively-stimulating games and toys, and novel experiences (e.g., trips to parks, museums, zoos, playgrounds, etc.) (54, 55). Enrichment can be manipulated in animal laboratory environments (56), making it particularly beneficial for the FXS field which has a strong set of animal models and suggesting the neurobiological mechanisms through which enrichment supports development in FXS can be directly interrogated. Like parental responsiveness, home enrichment is malleable and may serve as a key intervention target when working with families affected by FXS (53, 57).

### 4.4 Limitations and future directions

Several limitations of our study highlight directions for future research. First, we quantified peripheral FMRP as our measure of FMRP expression for both males and females with FXS. X-inactivation ratios provide one other method of characterizing molecular heterogeneity in female patients and may help account for additional phenotypic variance in females with FXS. Second, our findings are drawn from cross-sectional data, limiting insights into causality. Longitudinal work enabled by FXS natural history studies (i.e., FORWARD and FORWARD-MARCH) will help clarify causal pathways through which social-environmental factors impact development in FXS. Third, and relatedly, although the broad age range of our sample allowed us to study more individuals and maximize statistical power, this also limits the utility of some COI metrics such as the education subdomains (i.e., adults with FXS who no longer benefit from strong public education opportunities). Our use of concurrent addresses also deemphasizes the accumulative effect that limited access to social-environmental opportunities can have across the lifespan, beginning prenatally (58). However, participants in our study who moved between their visits typically remained in neighborhoods with similar levels of opportunity, suggesting that concurrent neighborhood resources may be similar to resources to which participants had access across their life. Retrospective studies of early childhood social-environmental exposures will clarify this accumulative effect. Fourth, consistent with national diagnostic and service access trends in FXS (42, 59), our sample was predominantly comprised of white, non-Hispanic patients. The neighborhoods for our sample also skewed towards higher opportunity areas (**Figure 1**). We have recently begun targeted efforts (e.g., in-home research visits) to minimize barriers to research participation, but this remains an ongoing need for the ID field more broadly. It is not known to what extent these findings generalize to a more racially and ethnically diverse sample or a sample more diverse in terms of neighborhood opportunity. Fifth, we have not yet incorporated other explanations of phenotypic variance into our study, including family genetics (e.g., as accounted for by a proxy like parental IQ) and epigenetic factors. These remain important areas for future study to comprehensively address factors driving individual differences in FXS. Sixth, we substituted ABIQ for FSIQ for the small number of participants who only completed the routing version of the SB-5. While we believe this is justified given findings demonstrating relatively strong ABIQ-FSIQ agreement when routing subtest scatter is minimal as in the present sample (28), this may introduce additional heterogeneity into our sample. Last, our interpretation of the COI assumes that a neighborhood’s level of resources (e.g., adequate insurance coverage) translates to an individual family (e.g., that family has adequate insurance coverage). The COI may minimize the role of individual experiences, and follow-up studies probing individual families’ social-environmental exposures will be necessary to translate these findings into actionable changes.

## 5 Conclusions

Our neighborhood-level analysis of social-environmental factors in FXS reflects the assessment of distal factors that affect development through multiple downstream, mediating pathways (e.g., neighborhood and family economic stability reduce family stress which promotes positive developmental outcomes). We see our use of the COI as a first step to generate hypotheses for future studies that will use narrower and more proximal social-environmental measures. These studies will allow individual families affected by FXS and other IDs to provide context on their own unique environments and the resulting impact on development. In addition to researchers incorporating these social-environmental measures, this study also highlights the need for clinicians to consider social determinants of health when caring for patients. Ultimately, we believe our findings highlight the complementary role that biological and environmental approaches play in understanding phenotypic variation in FXS and IDs more broadly.

## Data Availability

The raw data supporting the conclusions of this article will be made available by the authors, without undue reservation.
