# Supplementary material for: Beyond the Fragile X protein: neighborhood characteristics explain individual differences in IQ and adaptive behaviors of Fragile X syndrome
Source: Front Psychiatry. 2025 Sep 18;16:1636987. doi: 10.3389/fpsyt.2025.1636987 (PMC12488564; doi:10.3389/fpsyt.2025.1636987)
Supplement: Supplementary file 1 [file Table1.docx]

**SUPPLEMENTARY RESULTS**

***Associations between FMRP and IQ***

***Males***. Greater peripheral FMRP was associated with greater FSIQ (*F_(1,114.936)_* = 5.628, *p* = .019), VIQ (*F_(1,116.124)_* = 4.993, *p* = .027), and NVIQ (*F_(1,112.348)_* = 5.466, *p* = .021) in males.

***Females.*** Greater peripheral FMRP was associated with greater FSIQ (*F_(1,50.950)_* = 4.142, *p* = .047) and NVIQ (*F_(1,50.264)_* = 6.236, *p* = .016), but not VIQ (*F_(1,50.636)_* = 1.896, *p* = .175) in females.

***Associations between FMRP and adaptive behaviors***

***Males***. Greater peripheral FMRP was associated with greater overall adaptive behaviors (Vineland ABC; *F_(1,110.599)_* = 12.429, *p* < .001), adaptive communication skills (*F_(1,112.282)_* = 12.437, *p* < .001), adaptive social skills (*F_(1,110.914)_* = 6.104, *p* = .015), and daily living skills (*F_(1,108.699)_* = 13.943, *p* < .001).

***Females***. Greater peripheral FMRP was *not* associated with total adaptive behaviors (*F_(1,32.266)_* = 2.959, *p* = .095), adaptive communication skills (*F_(1,36.070)_* = 1.705, *p* = .200), adaptive social skills (*F_(1,31.678)_* = 3.028, *p* = .092), nor daily living skills (*F_(1,31.063)_* = 1.132, *p* = .296) in females.

***Associations between FMRP and neighborhood characteristics***

***Males***. Greater peripheral FMRP was not associated with any COI domains or subdomains in males.

***Females****.* Greater peripheral FMRP was associated with greater safety-related resources (*F_(1,57.583)_* = 4.638, *p* = .035) and greater neighborhood wealth (*F_(1,51.934)_* = 7.740, *p* = .008) in females.
